# Enhancing multivariate quality monitoring: Integrating LSTM and MLS-SVR with Max-MCUSUM frameworks

**DOI:** 10.1016/j.mex.2026.104197

**Published:** 2026-10-08

**Authors:** Muhammad Ahsan, Muhammad Romiz Rofan Hakim, Syafi’ Bariq’ Syihabuddin Hidayatullah, Muhammad Hisyam Lee

**Affiliations:** a Department of Statistic, Institut Teknologi Sepuluh Nopember, Surabaya, Indonesia; b Department of Mathematical Sciences, Universiti Teknologi Malaysia, Johor Bahru, Malaysia

**Keywords:** Control chart, LSTM, Max-MCUSUM, Quality control, Sugar production

## Abstract

This study enhances quality control in sugar production by monitoring solution color, grain size, and moisture content. These critical characteristics often exhibit autocorrelation, which can cause false alarms during statistical monitoring. To address this, we integrated Long Short-Term Memory (LSTM) and Multioutput Least-Squares Support Vector Regression (MLS-SVR) models with Max-MCUSUM control charts. The machine learning models were utilized to minimize Mean Squared Error (MSE) and extract residuals free of autocorrelation before applying Phase I and Phase II process monitoring. Results demonstrate that both LSTM and MLS-SVR successfully mitigated data autocorrelation. In the observed dataset, the LSTM-based Max-MCUSUM chart identified more Phase II out-of-control signals than the MLS-SVR-based chart. While Phase I established initial statistical control, Phase II detected three out-of-control signals primarily driven by variances in solution color and grain size. Despite these minor deviations, multivariate process capability indices (MCp and MCpk > 1) confirm the overall production reliably meets strict quality standards. Ultimately, leveraging LSTM or MLS-SVR residual-based Max-MCUSUM charts provides a highly effective framework for managing continuous production quality and pinpointing areas for process optimization.

## Highlights

• LSTM and MLS-SVR improve Max-MCUSUM monitoring by reducing autocorrelation in sugar quality data.
• The LSTM- and MLS-SVR-based charts produced different monitoring outcomes, with the LSTM-based chart identifying more out-of-control signals in the observed dataset.
• Multivariate process capability indices (MCp and MCpk > 1) confirm that the overall sugar production reliably meets strict quality standards despite minor process deviations.

## Graphical abstract

**Figure:**
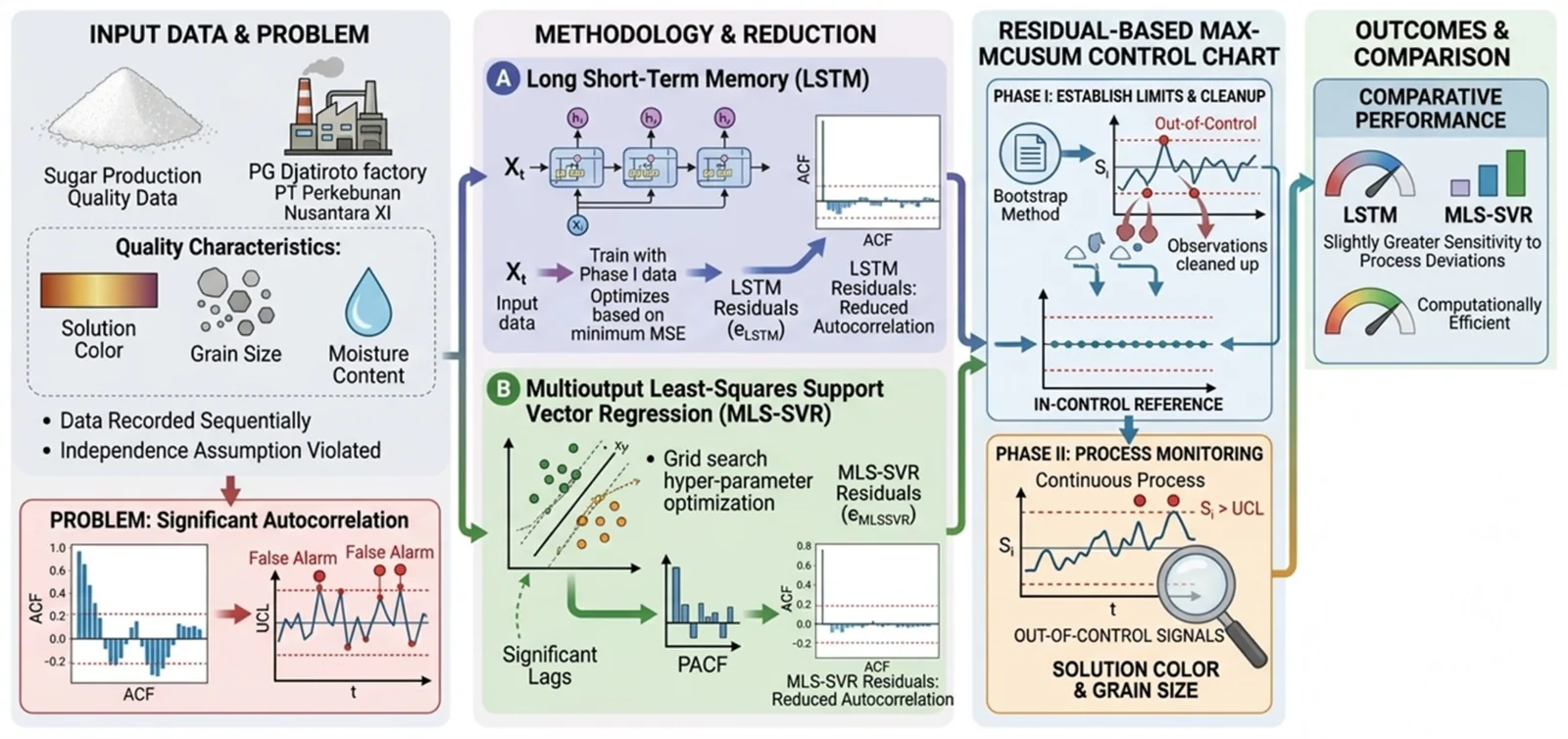

**Specifications table.**

This table provides general information on your method.

**Table:** 

**Subject area**	Mathematics and Statistics
**More specific subject area**	Statistical Process Control, Autocorrelation Data
**Name of your method**	Max-MCUSUM, LSTM, MLS-SVR
**Name and reference of original method**	1. **Max-MCUSUM** H. Khusna, M. Mashuri, M. Ahsan, S. Suhartono, and D. D. Prastyo, “Bootstrap-based maximum multivariate CUSUM control chart,” Qual. Technol. Quant. Manag., vol. 17, no. 1, pp. 52–74, Jan. 2020, doi: 10.1080/16843703.2018.1535765. 2. **LSTM** S. Hochreiter and J. Schmidhuber, “Long Short-Term Memory,” Neural Comput., vol. 9, no. 8, pp. 1735–1780, Nov. 1997, doi: 10.1162/neco.1997.9.8.1735. 3. **MLS-SVR** S. Xu, X. An, X. Qiao, L. Zhu, and L. Li, “Multi-output least-squares support vector regression machines,” Pattern Recognit. Lett., vol. 34, no. 9, pp. 1078–1084, Jul. 2013, doi: 10.1016/j.patrec.2013.01.015.
**Resource availability**	MATLAB, RStudio (R), and Python]

## Background

The sugar industry plays an important role in meeting national consumption needs, particularly for white crystal sugar, which is widely used for household consumption [1]. Based on the report of the Indonesian Sugar Association (AGI) and the Indonesian Sugar Experts Association (IKAGI), as published by Statistics Indonesia, white crystal sugar production decreased by 4.52% in 2020, from 2.2 million tons in 2019 to 2.13 million tons in 2020 [1]. This decline indicates the importance of quality control in the sugar production process to ensure that product quality remains in accordance with the required standards. Therefore, PG Djatiroto, as a sugar factory owned by PT Perkebunan Nusantara XI (PTPN XI), needs to apply an appropriate quality control method, one of which is the control chart.

In recent developments, control charts have not only been designed to monitor the process mean or variability separately, but have also been extended to monitor both parameters simultaneously in a single chart. The development of simultaneous control charts began with White and Schroeder (1987) [2], who introduced a simultaneous control chart for monitoring the process mean and variability in one chart. Chao and Cheng (1996) [3]combined the median and quartile deviation, while Chen and Cheng (1998) [4] developed the Max-Chart by combining the X and S charts. Furthermore, Khoo (2005) [5] proposed a bivariate control chart based on the combination of Hotelling’s T² and generalized variance (GV), which was later extended by Thaga and Gabaitiri (2006) [6] into the Multivariate Max-Chart (Max-Mchart). Further developments include the Gamma-transformation-based Max-Mchart [7], the half-normal transformation and bootstrap-based Max-Half-Mchart [8], and the extension of the Max-Mchart and Max-Half-Mchart for individual observations based on Hotelling’s T² and successive differences [9,10].

In addition to Shewhart-type charts, the development of simultaneous multivariate control charts has also been directed toward memory-type schemes, such as the Exponentially Weighted Moving Average (EWMA) and Cumulative Sum (CUSUM) charts. Memory-type control charts, including EWMA and CUSUM, are known to be more sensitive in detecting small to moderate shifts in process parameters. In the EWMA-type framework, several developments include omnibus EWMA schemes [11], the multivariate alternate variables chart proposed by Spiring and Cheng (1998) [12], the box chart proposed by Yeh and Lin (2002) [13], Max-EWMAMS [14], Max-DEWMA and single EWMA charts [15,16], Max-MEWMA [17], and generalized likelihood ratio-based EWMA charts for individual observations [18].

Meanwhile, in the CUSUM-type framework, several developments include Max-CUSUM and self-starting CUSUM charts [19,20], Max-CUSUM charts for autocorrelated data [21], Max-MCUSUM [22], and Max-MCUSUM charts for autocorrelated data [23]. Compared with Max-MEWMA, Max-MCUSUM has an advantage because it has been reported to provide better detection performance, particularly in the simultaneous monitoring of the process mean vector and covariance matrix. Cheng and Thaga (2005) [22] showed that Max-MCUSUM performs better than Max-MEWMA in multivariate process monitoring.

Therefore, Max-MCUSUM is relevant for this study because the quality characteristics of white crystal sugar are multivariate and the process deterioration may appear not only as a shift in the mean vector but also as a change in process variability. In addition, small to moderate shifts in production processes need to be detected as early as possible because delayed detection may lead to nonconforming product quality. Compared with Shewhart-type simultaneous charts, the CUSUM-based scheme accumulates information from previous observations, making it more sensitive to persistent small changes. Hence, the Max-MCUSUM chart provides an appropriate monitoring framework because it combines the advantages of the maximum-type statistic for simultaneous monitoring of mean and variability with the sensitivity of the CUSUM scheme for detecting small to moderate process shifts.

Quality control analysis using control charts generally requires the assumption that observations are independent over time [24]. However, in production process data recorded sequentially, this independence assumption is often not satisfied due to the presence of autocorrelation. Autocorrelation can affect the performance of control charts because it may increase false alarms or type I errors, resulting in less accurate decisions regarding the process condition [25,26]. One approach to reduce the effect of autocorrelation is to use residual-based control charts. This approach applies a time series model to capture the dependency pattern among observations, and the residuals from the model are then used as the input for the control chart because they are expected to be free from autocorrelation patterns [27].

In real applications, modeling autocorrelated data using traditional time series methods often faces limitations due to specific model assumptions and the complex dependency structure of the data. Support Vector Regression (SVR) can be used as an alternative method because it is capable of capturing both linear and nonlinear patterns [28,29]. A further development is Least Squares Support Vector Regression (LS-SVR), which simplifies the optimization process through a least-squares approach [30]. Multi-output Least Squares Support Vector Regression (MLS-SVR) was then developed to handle multi-output cases by modeling several responses simultaneously and considering the relationships among outputs [31,32]. In addition to MLS-SVR, Long Short-Term Memory (LSTM) can also be used as an alternative method because it is able to learn temporal patterns in sequential data through its memory and gate mechanisms [33].

Several previous studies have aimed to improve process and product quality by integrating statistical process control methods with machine learning techniques. Khusna et al. (2019) [34] developed an MLS-SVR residual-based Max-MCUSUM control chart to simultaneously monitor the mean and variability of autocorrelated multivariate processes. Furthermore, Yunika and Ahsan (2023) [35] applied an MLS-SVR residual-based Max-MCUSUM control chart to monitor the white crystal sugar production process at PG Djatiroto PTPN XI. In neural network-based approaches, Chen and Yu (2019) [36] proposed a residual control chart based on a deep Recurrent Neural Network (RNN) for autocorrelated processes. Other developments include the XGBoost regression residual-based MEWMA and MEWMV charts for water quality monitoring proposed by Sulistiawanti et al. (2023) [37], as well as the deep learning-based residual control chart for count data developed by Kim and Ha (2022) [38]. In addition, Aliyah and Ahsan (2025) [39] applied an LSTM residual-based Max-MCUSUM control chart to monitor autocorrelated water quality data.

While previous studies have successfully implemented residual-based control charts using individual machine learning or deep learning models, a direct comparative evaluation of these techniques within a simultaneous monitoring framework remains limited. To bridge this methodological gap, this study proposes a novel integration that juxtaposes a temporal memory-based architecture (LSTM) against a kernel-based multivariate regression model (MLS-SVR) directly within the Max-MCUSUM framework. This methodology distinctly advances existing literature by simultaneously monitoring both the process mean vector and the covariance matrix of highly autocorrelated data, extending beyond the conventional focus on isolated mean shifts. Furthermore, to address the multivariate non-normality frequently inherent in machine learning residuals, this framework incorporates bootstrap resampling to robustly estimate the upper control limits. By comparatively analyzing these two distinct predictive models within a unified, simultaneous CUSUM-based monitoring scheme, this research establishes a comprehensive empirical framework for enhancing detection sensitivity in complex, continuous industrial processes.

This study focuses on the application of quality control in the white crystal sugar production process using the Maximum Multivariate Cumulative Sum (Max-MCUSUM) control chart based on the residuals of Long Short-Term Memory (LSTM) and Multi-output Least Squares Support Vector Regression (MLS-SVR) models. This study also compares the monitoring results obtained from the LSTM and MLS-SVR residuals and evaluates the process capability of white crystal sugar production. The remainder of this paper is organized as follows. Section 2 presents the research materials and methods, including the white crystal sugar quality data, the Max-MCUSUM control chart, the LSTM model, and the MLS-SVR model. Section 3 presents the results and discussion, including data characteristics, autocorrelation analysis, residual-based Max-MCUSUM monitoring using LSTM and MLS-SVR, comparison of the two models, and process capability analysis. The final section discusses the limitations of the study and directions for future research.

## Method details

### Data collection

This research uses secondary data from the observation of white crystal sugar quality obtained from the quality control or quality assurance division of Djatiroto Sugar Factory PT Perkebunan Nusantara XI during the sugarcane milling season, namely 28 May 2021 - 25 October 2021 with Phase I from 28 May 2021 - 4 October 2021 and Phase II from 5 October 2021 - 25 October 2021. The dataset consisted of 130 observations in Phase I and 21 observations in Phase II. Because the two residual models use different temporal input structures, their effective sample sizes differ after input construction. Quality measurement is carried out by taking a 50-gram sample after the sugar passes through the vibrating screen, which the laboratory then processes. Several product quality characteristics determine the quality of white crystal sugar in this research using three quality characteristics: solution color, grain size, and moisture content.

### Data preprocessing

Before further analysis, a dependency test was conducted to evaluate the relationship among the three quality characteristics using Bartlett’s value. In addition, the Autocorrelation Function (ACF) and Partial Autocorrelation Function (PACF) plots were examined to identify the autocorrelation pattern and determine significant lags in the data. The significant lags were then used as inputs for the LSTM and MLSSVR models to capture time-dependent patterns in the sugar quality data.

### Model development

#### Maximum multivariate cumulative sum (Max-MCUSUM)

There are several cases where several correlated quality characteristics determine the quality of a product; some researchers proposed a multivariate Control Chart to monitor a process, which was later developed into a Max-MCUSUM Control Chart to monitor product quality in the average and variability of the process in one diagram [8]. Several assumptions must be met to ensure the effectiveness of using Max-MCUSUM control charts, including that the data must be normally distributed and the observations must be mutually independent. The assumption of normally distributed data is critical to maintaining the accuracy of the Max-MCUSUM control chart. However, the advantage of the Max-MCUSUM control chart is that it is relatively robust to the normal distribution assumption. If the data do not fulfill the assumption of multivariate normal distribution, the Max-MCUSUM control chart can still be used while providing accurate results. However, if the assumption of mutual independence is not met, this can interfere with the performance of the Max-MCUSUM control chart. This is because autocorrelated data will cause many false alarms.

(1) $$Z_{i}=\frac{{\left(\mu_{e\left(b\right)}-\mu_{e\left(g\right)}\right)}^{T}\sum_{e\left(g\right)}^{-1}\left(e_{i}-\mu_{e\left(g\right)}\right)}{{\left(\mu_{e\left(b\right)}-\mu_{e\left(g\right)}\right)}^{T}\sum_{e\left(g\right)}^{-1}{\left(\mu_{e\left(b\right)}-\mu_{e\left(g\right)}\right)}^{\frac{1}{2}}}$$

where $\mu_{e(b)}\;$denotes the mean vector of residuals under the out-of-control (shifted) condition, $\mu_{e(g)}\;$denotes the mean vector of residuals under the in-control condition, and $\Sigma_{e(g)}\;$denotes the covariance matrix of the in-control residuals. The vector $\mu_{e(b)\;}$represents the mean vector associated with the shifted or out-of-control residual condition, whereas $e_{i}\;$denotes the individual residual vector being monitored. If the process residual vector $e_{i}$ is in-control, then the mean vector $\mu_{e(g)}\;$and the random variable $Z_{i}$ are standard normal distributed. And if not, then $Z_{i}$ follows a normal distribution with mean λ. The non-centrality parameter λ can be calculated as follows:

(2) $$\lambda ={\left[{\left(\mu_{e\left(b\right)}-\mu_{e\left(g\right)}\right)}^{T}\sum_{e\left(g\right)}^{-1}\left(\mu_{e\left(b\right)}-\mu_{e\left(g\right)}\right)\right]}^{\frac{1}{2}}$$

When the residual vector $e_{i}$ is obtained from the shifted process, then the vector experiences a shift in the covariance matrix $\sum_{e}\left(b\right)$, it can be written as the following equation.

(3) $$W_{i}=\Phi^{-1}\left\{H\left[{\left(\mu_{e\left(b\right)}-\mu_{e\left(g\right)}\right)}^{T}\sum_{e\left(b\right)}^{-1}\left(e_{i}-\mu_{e\left(g\right)}\right);m\right]\right\}$$

Where Φ(z)=P(Z≤z) with Z∼N(0.1). The function $\Phi^{-1}$ is symbolized as the inverse of the cumulative function of the standard normal distribution, and H(x;m)=P(X≤x|m) with $X∼X_{m}^{2}$. The random variables $Z_{i}$ and $W_{i}$ are independent and follow a normal distribution. So, the following is the statistics of the Max-MCUSUM Control Chart equation as follows [23].

(4) $$M_{\left(r\right)i}=\text{max}\left[C_{\left(r\right)i},S_{\left(r\right)i}\right]$$

Where $C_{(r)i}\;$is the transformation of the random variable $Z_{i}$ and $S_{(r)i}\;$is the transformation of the random variable $W_{i}$, which can be written in the following equation.

(5) $$\begin{array}{c} C_{\left(r\right)i}=max\left[C_{\left(r\right)i}^{+},C_{\left(r\right)i}^{-}\right]\\ C_{\left(r\right)i}^{+}=max\left[0,Z_{i}-k+C_{\left(r\right)i-1}^{+}\right]\\ C_{\left(r\right)i}^{-}=max\left[0,-Z_{i}-k+C_{\left(r\right)i-1}^{-}\right] \end{array}$$

(6) $$\begin{array}{c} S_{\left(r\right)i}=max\left[S_{\left(r\right)i}^{+},S_{\left(r\right)i}^{-}\right]\\ S_{\left(r\right)i}^{+}=max\left[0,W_{i}-k+S_{\left(r\right)i-1}^{+}\right]\\ S_{\left(r\right)i}^{-}=max\left[0,-W_{i}-k+S_{\left(r\right)i-1}^{-}\right] \end{array}$$

Given $C_{(r)0}$ and $S_{(r)0}$ are the starting point and reference value k > 0 determined at the first time of monitoring. in this study, k=0.5was adopted following the bootstrap-based Max-MCUSUM framework of Khusna et al. [34] and the same value was retained for Phase II monitoring. The statistics of the Max-MCUSUM Control Chart can only be compared with the UCL, when $M_{(r)i}$ ≥ 0. That way, it can detect OC signals when $M_{i}$ is more than the UCL. The calculation for the upper control limit of the Max-MCUSUM Control Chart can be estimated using the bootstrap method, which is one of the resampling methods for estimating parameters with unknown distributions; the equation can be written as follows.

(7) $$\text{UCL}=\frac{1}{N^{\prime }}\sum_{l=1}^{N^{\prime }}M_{\left(r\right)\left(100\left(1-\alpha \right)\right)}^{l}$$

Where $M_{\left(r)(100(1-\alpha )\right)}^{l}=M_{\left((1-\alpha )B\right)}^{l}$ and $l=1,2,\ldots ,N^{\prime }$. In this study, $N^{\prime }=1000$ replications were performed. Within each replication, B=1000Max-MCUSUM statistics were resampled with replacement. The upper control limit was determined as the 100(1−α)-th percentile of the resulting bootstrap distribution.

#### Long short-term memory (LSTM)

Recurrent Neural Network (RNN) algorithm is a part of deep learning that processes sequential data. RNN is a model that mimics how humans make decisions by not discarding information from the past in the learning process. In RNN, information from the past is automatically stored and considered when processing inputs. RNNs work by processing sequential data, meaning each sample is processed in a specific order, and each sample in the sequence has a close relationship. To optimize RNN performance, various types of architectures can be used, one of which is Long Short-Term Memory (LSTM). RNNs are often used in speech, natural language modeling, and handwriting recognition. A Cost Function equation is used to compare the prediction results or output values generated with the target or the actual value. The Cost Function equation used in this research is Mean Squared Error (MSE). Mean Squared Error (MSE) is one cost function used to measure the difference between model predictions and actual values. MSE calculates the average square of the difference between the prediction and the true value for each data sample. If the MSE value is smaller, the model performs better in making predictions. In addition to MSE, other types of cost functions, such as cross-entropy loss and hinge loss, are suitable for use depending on the problem and model used in the equation [40].

(8) $$E=\frac{1}{n}\sum_{i=1}^{n}{\left(y_{i}-z_{i}\right)}^{2}$$

where E denotes the MSE error or cost function, yis the target vector, zis the output vector generated by the model, and nis the number of observations.

Long-Short-Term Memory (LSTM) is a neural network model developed to overcome the long-term dependency problem in Recurrent Neural Networks (RNN). This problem occurs because RNN only uses one simple layer that produces an output based on the current input and the previous step's production. The layer used in the looping RNN network is the tanh layer. The equation for the LSTM method is described in the following equation [33].

(9) $$\begin{array}{c} f_{t}=\sigma \left(W_{f}\times \left[h_{t-1},y_{t}\right]+b_{f}\right)\\ i_{t}=\sigma \left(W_{i}\times \left[h_{t-1},y_{t}\right]+b_{i}\right)\\ \bar{C}=\tanh\left(W_{c}\times \left[h_{t-1},y_{t}\right]+b_{c}\right)\\ C_{t}=f_{t}\times C_{t-1}+i_{t}\times \bar{C}\\ o_{t}=\sigma \left(W_{o}\times \left[h_{t-1},y_{t}\right]+b_{o}\right)\\ h_{t}=o_{t}\times \tanh\left(C_{t}\right) \end{array}$$

#### Multioutput least square support vector regression (MLS-SVR)

Least Square Support Vector Regression (LS-SVR) learns about mapping from an input to a single output. If a case solved by the LS-SVR algorithm has multi-outputs, the output cross-linkages are ignored. Single-output regression can be generalized to multi-output regression [31]. MLS-SVR algorithm with kernel function mapping from d dimension to h dimensions, where h is Hilbert space, a higher dimension. The MLS-SVR function is obtained using the following equation [34].

(10) $$\begin{array}{c} \hat{f}\left(x\right)=\varphi {\left(x\right)}^{T}\tilde{W}+{\tilde{b}}^{T}\\ =\varphi {\left(x\right)}^{T}repmat\left({\tilde{w}}_{0},1,m\right)+\varphi {\left(x\right)}^{T}\tilde{V}+{\tilde{b}}^{T}\\ =\varphi {\left(x\right)}^{T}repmat\left(\sum_{j=1}^{m}Z{\tilde{\alpha }}_{j},\;1,\;m\right)+\frac{m}{\gamma^{\prime \prime }}\varphi {\left(x\right)}^{T}Z{\tilde{A}}^{{}^{\prime }}+{\tilde{b}}^{T}\\ =repmat\left(\sum_{j=1}^{m}\sum_{i=1}^{n}{\tilde{\alpha }}_{\text{ij}}\tilde{K}\left(x,x_{i}\right),\;1,\;m\right)+\frac{m}{\gamma^{\prime \prime }}\sum_{i=1}^{n}{\tilde{\alpha }}_{i}\tilde{K}\left(x,x_{i}\right)+{\tilde{b}}^{T}. \end{array}$$

The kernel function used is the Radial Basis Function (RBF), which is used to implement the model in a higher dimension without defining the mapping function of the dimension without having to define a mapping function from the dimension to the higher dimension. The kernel function can be written with the following equation [31].

(11) $$K\left(x,x_{i}\right)=\text{exp}\left(-\sigma {\left|\left|x-x_{i}\right|\right|}^{2}\right)$$

Furthermore, the hyper-parameters of the MLS-SVR model will be determined using the grid search method. The algorithm of this method is to make grid points that have the same distance, which have the same distance, which can be calculated as the size of the error of each parameter point. To ensure a sufficiently broad and consistent search space, the same exponential range was considered for all three hyperparameters: $\gamma^{\prime }\in \left\{2^{-10},2^{-8},\ldots ,2^{20}\right\}$, $\gamma^{\prime \prime }\in \left\{2^{-10},2^{-8},\ldots ,2^{20}\right\}$, and $\sigma \in \{2^{-10},2^{-8},\ldots ,2^{20}\}$, where σdenotes the RBF kernel parameter. A total of 4,096 parameter combinations were evaluated, and the combination yielding the minimum MSE was selected.

### Analysis steps

The analytical framework applied in this study consists of the following steps.

1. Problem Definition and Data Collection: Define the quality monitoring issue in the white crystal sugar production process at PG Djatiroto and collect the relevant quality characteristics, including solution color, grain size, and moisture content.
2. Data Partitioning: Divide the dataset into Phase I data as the initial in-control phase and Phase II data as the monitoring phase.
3. Data Characteristics and Dependency Analysis: Conduct descriptive statistical analysis for each quality characteristic. Assess the dependency among the three quality characteristics using Bartlett’s test to ensure the suitability of multivariate monitoring.
4. Autocorrelation Analysis: Examine the presence of autocorrelation using the Autocorrelation Function (ACF) and Partial Autocorrelation Function (PACF) plots. Identify significant lag patterns to determine the temporal dependencies that need to be modeled before applying the control chart.
5. LSTM and MLS-SVR Modeling: Develop LSTM and MLS-SVR models to capture temporal dependencies in the sugar quality data. Apply min–max scaling to the LSTM inputs using Phase I data, whereas the MLS-SVR inputs are constructed from significant PACF-based lagged observations without min–max scaling. Optimize the model architecture and hyperparameters based on the minimum Mean Squared Error (MSE). The fitted Phase I models are then applied to the Phase II data without refitting to obtain Phase II predictions and residuals.
6. Residual Analysis: Calculate residuals from the difference between the actual and predicted values produced by the LSTM and MLS-SVR models. Evaluate the residuals using ACF plots and multivariate normality testing. Multivariate normality was assessed at a significance level of 0.05 using the multivariate Shapiro–Wilk test.
7. Max-MCUSUM Control Chart: Apply the residual-based Max-MCUSUM control chart to Phase I data to establish the control limit. Use the obtained control limit to monitor Phase II residuals and detect out-of-control signals.
8. Identification of Out-of-Control Causes: Identify the quality characteristics contributing to out-of-control signals by analyzing combinations of solution color, grain size, and moisture content.
9. Model Comparison: Compare the performance of LSTM and MLS-SVR based on their ability to reduce autocorrelation, prediction accuracy, sensitivity in detecting process shifts, and computational efficiency.
10. Process Capability Analysis and Conclusion: Evaluate process capability using Cp, Cpk, MCp, and MCpk indices. Summarize the findings and provide recommendations for improving the white crystal sugar production quality control process.

### Pseudocode

Input:

X_t = multivariate time series data for t = 1 to T

X_t = [solution color_t, grain size_t, moisture content_t]

Phase_I_range = time range for initial control phase

Phase_II_range = time range for process monitoring phase

k = reference value of Max-MCUSUM

alpha = significance level for control limit

B = number of bootstrap samples

Output:

Out-of-control signals from residual-based Max-MCUSUM chart

Comparison results between LSTM and MLS-SVR models

Process capability indices Cp, Cpk, MCp, and MCpk

Procedure:

Step 1: Define Data and Quality Characteristics

Collect white crystal sugar quality data

Select three quality characteristics:

solution color, grain size, and moisture content

Step 2: Data Partitioning

Divide X_t into:

Phase_I = data for establishing control limits

Phase_II = data for monitoring the process

Step 3: Descriptive and Dependency Analysis

Calculate descriptive statistics for each quality characteristic

Perform Bartlett’s test to evaluate dependency among variables

Step 4: Autocorrelation Checking

For each quality characteristic:

Plot ACF and PACF

Identify the presence of autocorrelation

Identify significant lag patterns

If autocorrelation exists:

Proceed to residual modeling

Else:

Proceed directly to Max-MCUSUM chart

Step 5: Build LSTM Model

Fit the min-max scaler using Phase_I data

Transform Phase_I data using the fitted scaler

Train and optimize the LSTM model using Phase_I data

Select the architecture and hyperparameters based on minimum MSE

Generate Phase_I predicted values

Calculate Phase_I residuals:

e_LSTM_PhaseI[t] = X_actual[t] - X_pred_LSTM[t]

Apply the fitted Phase_I scaler to Phase_II data

Apply the fitted Phase_I LSTM model to Phase_II data without refitting

Generate Phase_II predicted values

Calculate Phase_II residuals:

e_LSTM_PhaseII[t] = X_actual[t] - X_pred_LSTM[t]

Step 6: Build MLS-SVR Model

Identify significant lags using the Phase_I PACF plot

Form MLS-SVR input variables based on the selected lags

Train and optimize the MLS-SVR model using Phase_I data

Select the hyperparameters using grid search based on minimum MSE

Generate Phase_I predicted values

Calculate Phase_I residuals:

e_MLSSVR_PhaseI[t] = X_actual[t] - X_pred_MLSSVR[t]

Apply the fitted Phase_I MLS-SVR model to Phase_II data without refitting

Generate Phase_II predicted values Calculate Phase_II residuals:

e_MLSSVR_PhaseII[t] = X_actual[t] - X_pred_MLSSVR[t]

Step 7: Residual Validation

For each residual model:

Check autocorrelation using ACF plot

Test multivariate normality

If residual autocorrelation is reduced:

Proceed to Max-MCUSUM chart

Else:

Rebuild the model and adjust hyperparameters

Step 8: Build Max-MCUSUM Chart for Phase I

For each model residual:

Initialize Max-MCUSUM statistic

Calculate Max-MCUSUM statistic for Phase_I residuals(continued)

Determine UCL using bootstrap method

If S_t > UCL:

Signal out-of-control at time t

For LSTM:

Replace the corresponding residual with the Phase I mean residual

For MLS-SVR:

Replace the corresponding residual with the previous in-control residual

Recalculate the Max-MCUSUM statistic and UCL

Repeat until Phase I is statistically in control

Step 9: Monitor Phase II

For each model residual in Phase_II:

Calculate Max-MCUSUM statistic using Phase_I control limit

If S_t > UCL:

Signal out-of-control at time t

Else:

Process is statistically in control at time t

Step 10: Identify Out-of-Control Contributors

Form combinations of quality characteristics:

solution color and grain size

solution color and moisture content

grain size and moisture content

Apply Max-MCUSUM chart to each combination

Identify the combination with the highest number of out-of-control signals

Step 11: Compare LSTM and MLS-SVR

Compare both models based on:

MSE value

reduction of autocorrelation

number of out-of-control signals

sensitivity to process shifts

computation time

Step 12: Process Capability Analysis

Calculate univariate capability indices:

Cp and Cpk

Calculate multivariate capability indices:

MCp and MCpk

End Procedure

### Model evaluation

Before further analysis, a dependency test will be conducted to evaluate the relationship between the three quality characteristics using Bartlett's value of 8.300 and the degree of freedom of 3, so the p-value is 0.040. Based on these results, it can be concluded that the variables in this data are significant. The theory in the field also reinforces this: the three quality characteristics are related. When the water content of white crystal sugar is higher, the grain size will be greater, and when the grain size is greater, the color of the sugar solution formed will be brown.

Some assumptions must be fulfilled in this research, including that there is no autocorrelation or each data observation is independent. Before checking for autocorrelation, the first step is standardizing the three quality characteristics. Therefore, to check for autocorrelation, we look at each sugar quality characteristic's ACF (Autocorrelation Function) plot, as shown in Fig. 1

**Fig. 1:**
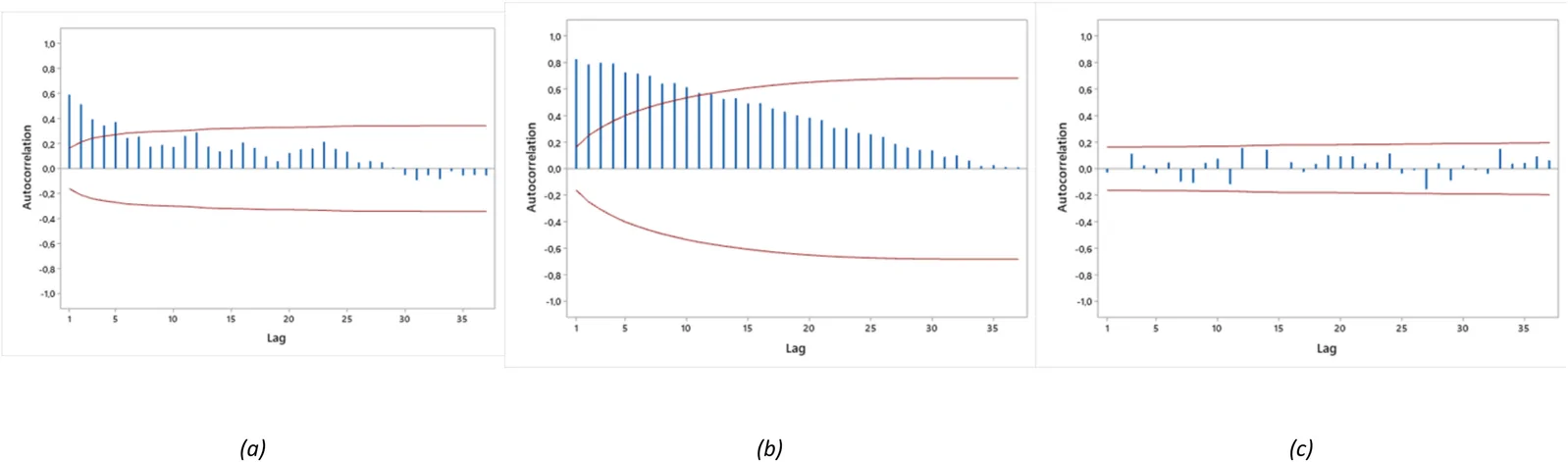
(a) ACF plot of solution color; (b) ACF plot of grain size; (c) ACF plot of moisture content.

Fig. 1 shows that the three quality characteristics of white crystal sugar in the Djatiroto sugar factory have significant autocorrelation. The presence of autocorrelation in the sugar production process observation data is due to the continuous nature of the production process. So, to overcome the autocorrelation in this observation data, LSTM and MLS-SVR modeling approaches are used. This approach helps overcome the effects of autocorrelation in the observational data by explaining the analysis results using both models.

### Data modeling

#### Modeling using LSTM

LSTM modeling determines the best architecture and hyper-parameters by trial and error to get the residual results of the LSTM model with the lowest MSE value calculation characteristics. The architecture and hyperparameters obtained from Phase 1 will be used for the Phase 2. The LSTM input data were transformed using min–max scaling. The scaler was fitted using the Phase I training data only, and the same fitted scaling parameters were subsequently applied to the Phase II data to avoid information leakage. The scaled data were then organized into input–target pairs using a one-step sliding window (lookback window = 1), where the multivariate observation at time t was used to predict the observation at time t+1. Consequently, the 130 observations in Phase I produced 129 effective LSTM input–target pairs, while the 21 observations in Phase II produced 20 effective pairs. The calculation results obtained the best LSTM model architecture and hyperparameters, as shown in Table 1.

**Table 1:** Architecture and hyper-parameters LSTM.

Layer	Hyperparameter
LSTM Layer 1	Units: 512; Recurrent Dropout: 0.2; Kernel Regularizer: l2(0.01)
Batch Normalization	-
Dropout	Rate:0.4
Skip Connection 1	LSTM Units: 256; Recurrent Dropout: 0.3; Kernel Regularizer: l2(0.01)
Skip Connection 1 Residual	LSTM Units: 256; Recurrent Dropout: 0.3; Kernel Regularizer: l2(0.01)
Skip Connection 2	LSTM Units: 128; Recurrent Dropout: 0.3; Kernel Regularizer: l2(0.01)
Skip Connection 2 Residual	LSTM Units: 128; Recurrent Dropout: 0.3; Kernel Regularizer: l2(0.01)
Skip Connection 3	LSTM Units: 64; Recurrent Dropout: 0.3; Kernel Regularizer: l2(0.01)
Skip Connection 3 Residual	LSTM Units: 64; Recurrent Dropout: 0.3; Kernel Regularizer: l2(0.01)
Skip Connection 4	LSTM Units: 32; Recurrent Dropout: 0.3; Kernel Regularizer: l2(0.01)
Skip Connection 4 Residual	LSTM Units: 32; Recurrent Dropout: 0.3; Kernel Regularizer: l2(0.01)
Final LSTM Layer	Units: 32; Recurrent Dropout: 0.3; Kernel Regularizer: l2(0.02)
Batch Normalization	-
Dropout	Rate: 0.4
Dense Layer 1	Units: 256; Activation: leaky relu
Batch Normalization	-
Dense Layer 2	Units: 128; Activation: leaky relu
Batch Normalization	-
Dense Layer 3	Units: 64; Activation: leaky relu
Batch Normalization	-
Dense Layer 4	Units: 32; Activation: swish
Batch Normalization	-
Final Dense Layer	Activation: linear

Therefore, the Nesterov-accelerated Adaptive Moment Estimation (Nadam) optimizer is used with a learning rate of 0.001 and some additional parameters such as $\beta_{1},\;\beta_{2},\in$ and schedule decay. This step sets how the model will minimize the loss function during training. After that, the model was trained using the fit method with the number of epochs set at 200 and batch size at 8. During training, an early stopping callback is used to monitor the loss on the validation set and stop training if there is no significant improvement. The entire training and model evaluation process is recorded in the history variable, which will contain training metrics such as loss and validation metrics such as validation loss for each epoch.

Based on the calculation results with training data, the MSE value is 0.115, indicating that the average square of the difference between the predicted and actual values is relatively small. Overall, the LSTM model has produced satisfactory and reliable results in making predictions. After modeling using LSTM in phase 1, the predicted value of Y is compared with the actual data value which can be seen in Fig. 2.

**Fig. 2:**
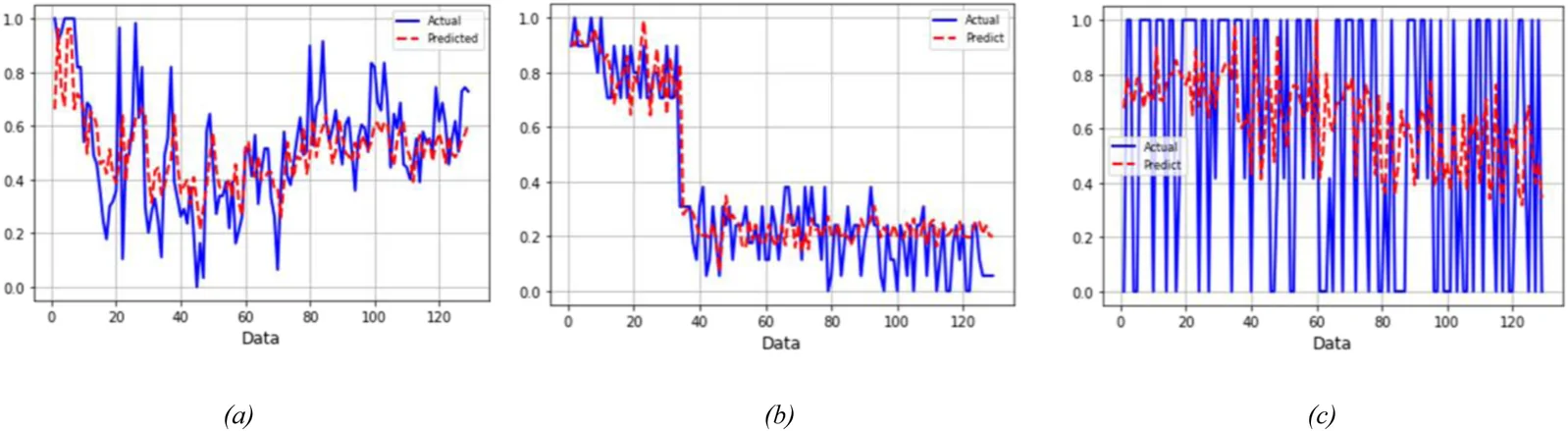
(a) Line plot of actual vs. predicted phase I solution color; (b) line plot of actual vs predicted phase I grain size; (c) line plot of actual vs. predicted phase I moisture content.

Fig. 2 shows line plots of the three sugar characteristics, showing that the predicted value data from LSTM modeling has a similar pattern to the actual phase I data value. This indicates that LSTM modeling can be used because the prediction data pattern tends to be the same as the actual data. The LSTM model fitted in Phase I was subsequently applied to the Phase II data without refitting or updating the model parameters, as shown in Fig. 3.

**Fig. 3:**
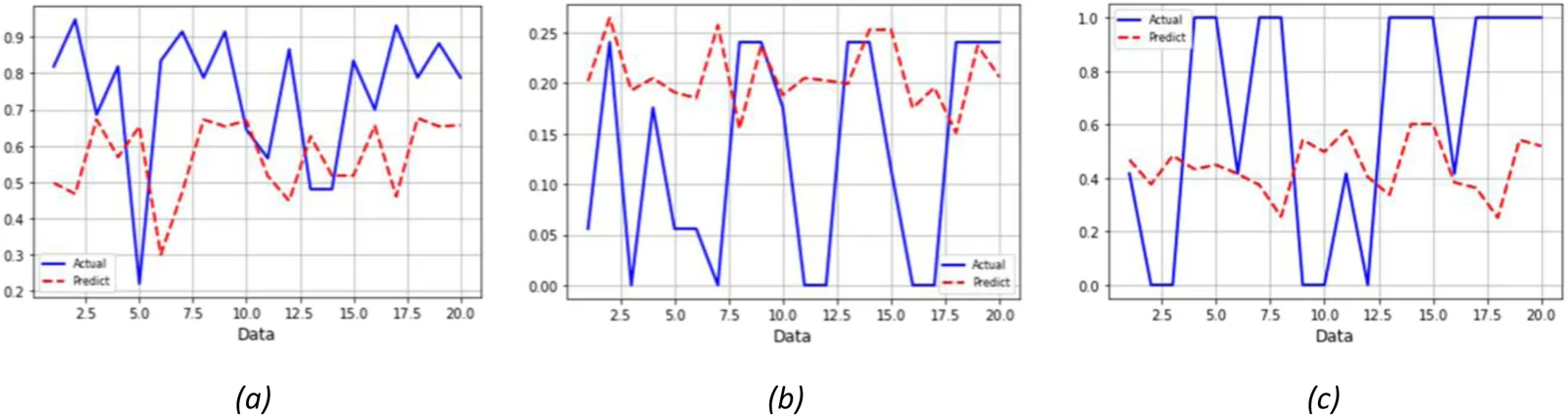
(a) Line plot of actual vs predicted phase II solution color; (b) line plot of actual vs predicted phase II grain size; (c) line plot of actual vs predicted phase II moisture content.

The line plots comparing the predicted and actual values of the three white crystal sugar quality characteristics in Phase II show noticeable differences in their patterns. Therefore, the residuals from the LSTM model were further evaluated for autocorrelation before being monitored using the Max-MCUSUM control chart. The Phase I residual ACF plots in Fig. 4 were used to evaluate whether the LSTM model reduced autocorrelation.

**Fig. 4:**
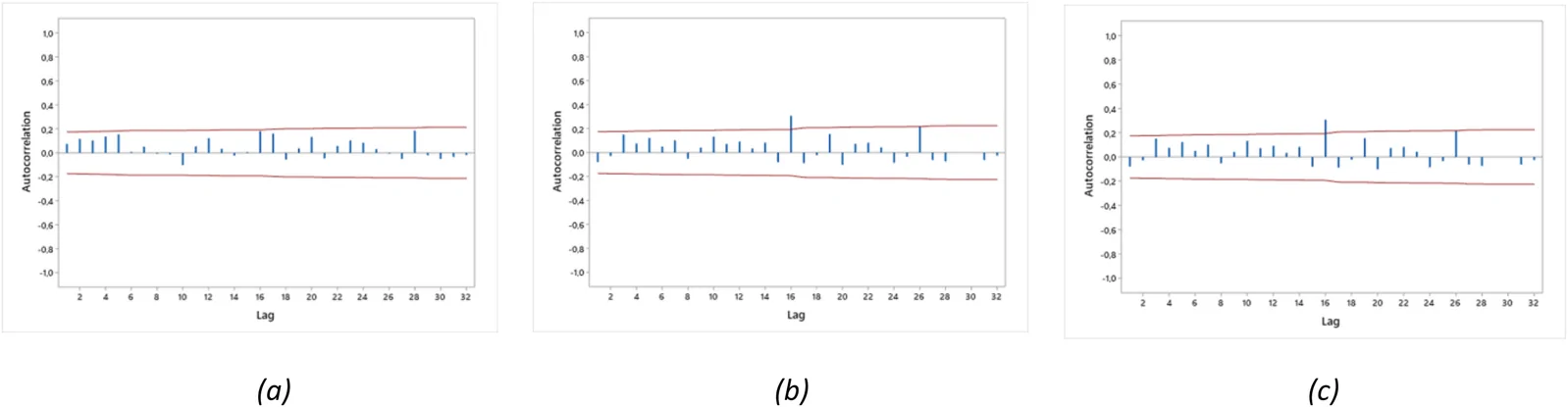
(a) ACF plot of solution color; (b) ACF plot of grain size; (c) ACF plot of moisture content.

The ACF plots of the three quality characteristics above show that LSTM modeling can reduce autocorrelation in the phase I observation data. For practical implementation, residual autocorrelation is considered acceptable when most ACF values lie within the 95% confidence bounds and no persistent pattern appears across consecutive lags. Further model refinement is required when several consecutive lags remain significant or a systematic autocorrelation pattern is still observed. After checking for autocorrelation in the residual data of the phase I LSTM model, a multivariate normal distribution check was performed on the three quality characteristics of white crystal sugar. The multivariate normal test was carried out using the multivariate Shapiro–Wilk test with the results obtained by the W value of 0.961 so that the p-value was $2.721e^{-08}$. Due to the p-value < α, it can be concluded that the assumption of multivariate normal distribution in this study has not been fulfilled. However, the quality control method with the Max-MCUSUM Control Chart is robust to the assumption of multivariate non-normal distribution so that this research can continue. After conducting assumption testing on the LSTM model residual data, the data is used in the MAX-MCUSUM Control Chart, which is a more effective method because it can simultaneously monitor the average and variability of the process. The control limits on the bootstrap-based Max-MCUSUM Control Chart have been evaluated using the ARL criterion. ARL is the average number of observations until the first observation is detected out of control. This Control Chart is used to detect small process shifts that may occur with the ARL_0_ value used in this study, which is 370 with α of 0.00273.

#### Phase-I

Fig. 5 (a) shows that the control limit in phase I is 3.334 with k = 0.5 and α = 0.00273; two observations are outside the control limit, namely the 22nd and 34th observation data, which means that the residuals of the LSTM model of the PG Djatiroto white crystal sugar production process in the 2021 milling season in phase I are still not statistically controlled and cannot be said to have represented an in-control process due to shifting process variability due to the statistical value of S_i_ > UCL. Out-of-control signals caused by process variability so to overcome out-of-control observation data by replacing the residual value of the observation data with the average residual value. The residual-based Max-MCUSUM Control Chart of the LSTM phase I model that has been statistically controlled is shown in Fig. 5 (b). Therefore, the process can proceed to phase II with a control limit of 3.334 to monitor process shifts in white crystal sugar production at PG Djatiroto in the 2021 milling season phase II.

**Fig. 5:**
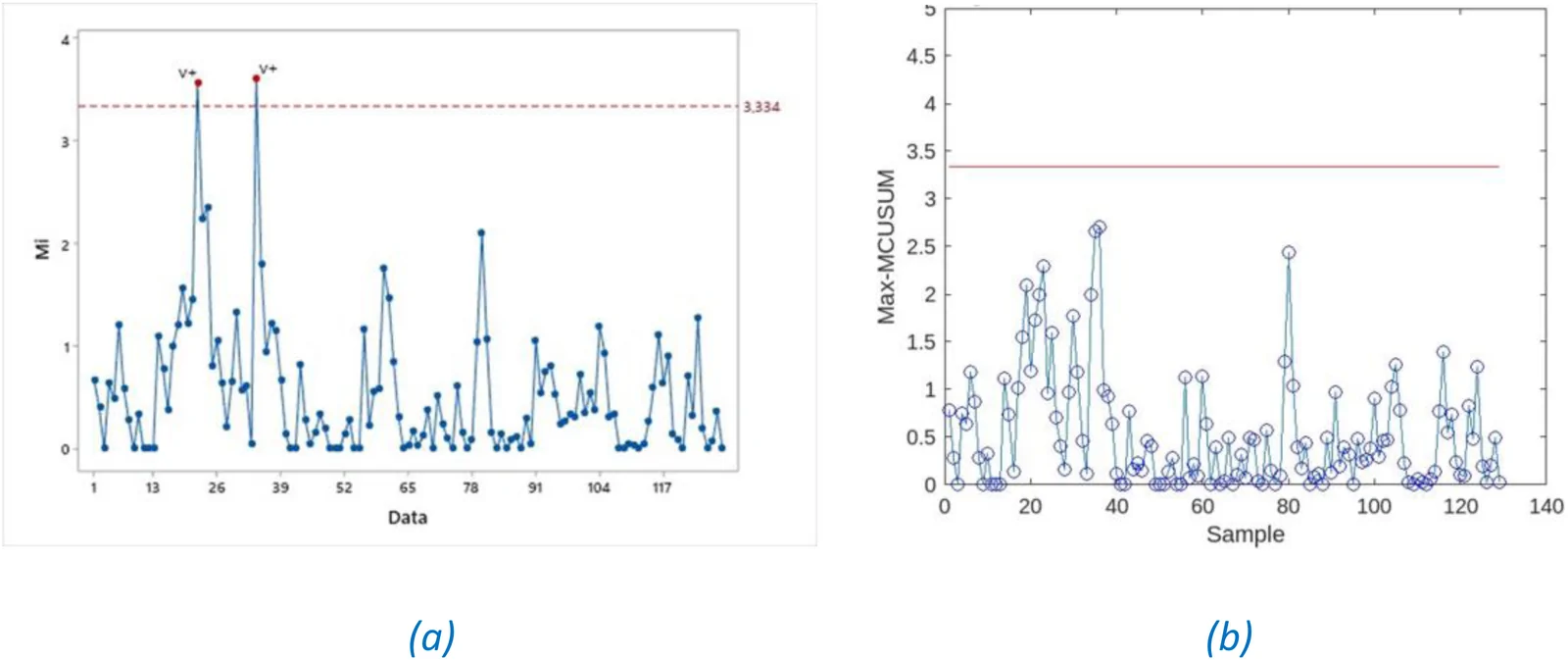
(a) Max-MCUSUM chart based on LSTM residuals phase I; (b) Max-MCUSUM chart based on LSTM residuals phase I in control.

#### Phase-II

Based on Fig. 6, the phase II Max-MCUSUM Control Chart with k = 0.5 and control limits of 3.334 three observation points are outside the control limits. So, it can be said that the sugar yield production process at Djatiroto Sugar Factory PT Perkebunan Nusantara XI has not been statistically controlled and the average process has shifted. The analysis showed that the production process was not statistically controlled in phases I and II. Therefore, the cause of OOC was identified by considering the combination of two quality characteristic variables: solution color and grain size, solution color and moisture content, and grain size and moisture content. This identification was done using the Max-MCUSUM Control Chart with parameter k = 0.5. The results of out-of-control identification from the Max-MCUSUM Control Chart using the combination of each quality characteristic can be shown in Table 2.

**Fig. 6:**
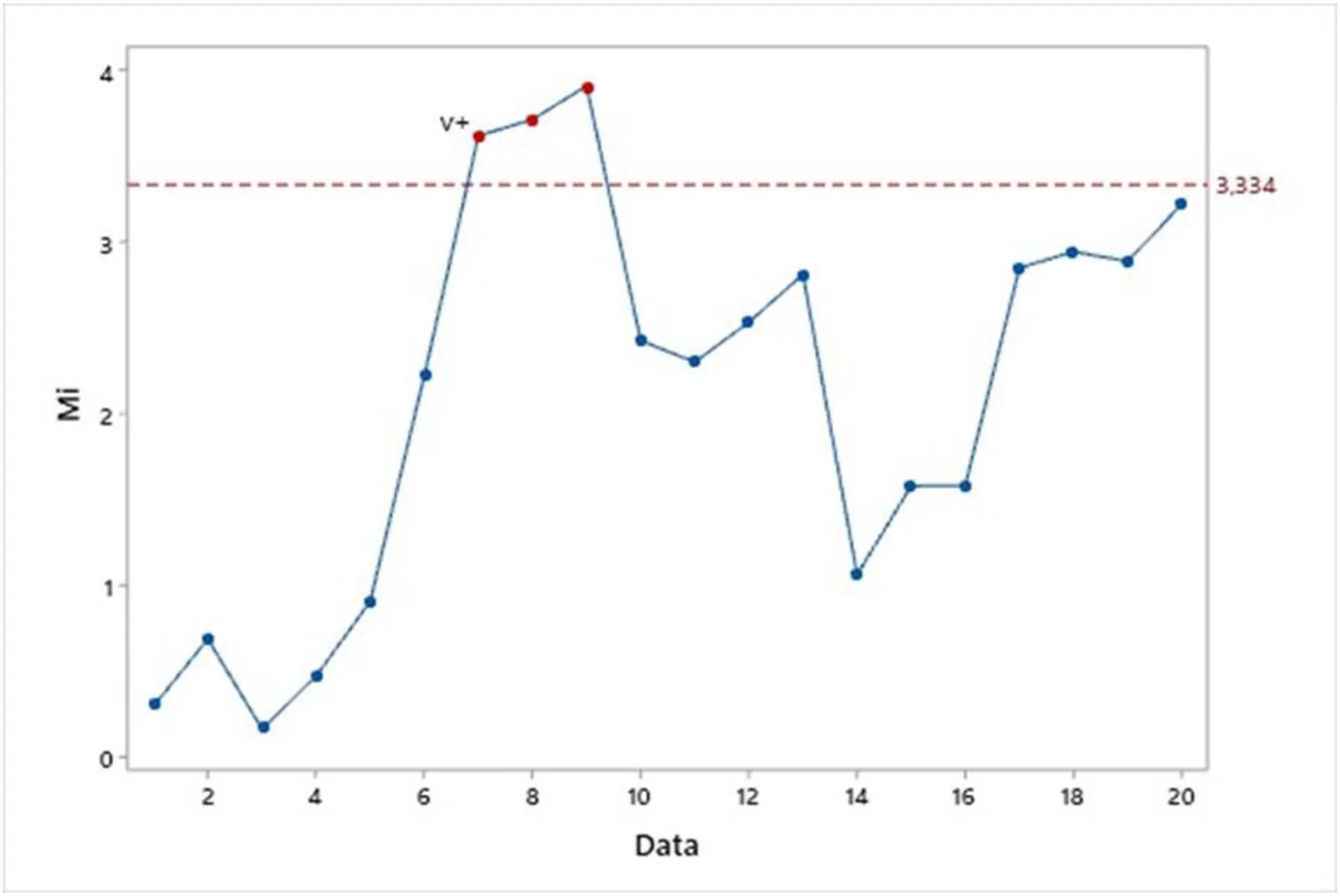
Max-MCUSUM chart based on LSTM residuals phase II.

**Table 2:** Phase I out-of-control identification results for quality-characteristic combinations using the LSTM-based Max-MCUSUM chart.

Combination of Characteristics Quality	Description	Many Observations Out of Control
Solution Color, Grain Size, Moisture Content	Out-of-control	2
Solution Color and Grain Size	Out-of-control	3
Solution Color and Moisture Content	Out-of-control	1
Grain Size and Moisture Content	Out-of-control	2

Following the Phase II monitoring results, the out-of-control signal previously identified during Phase I was further investigated by examining combinations of the quality characteristics. The identification results for the MLS-SVR-based Max-MCUSUM chart are presented in Table 3.

**Table 3:** Phase I out-of-control identification results for quality-characteristic combinations using the MLS-SVR-based Max-MCUSUM chart.

Combination of Characteristics Quality	Description	Many Observations Out of Control
Solution Color, Grain Size, Moisture Content	out	1
Solution Color and Grain Size	out	3
Solution Color and Moisture Content	out	3
Grain Size and Moisture Content	out	3

Table 2 shows the out-of-control identification results for different combinations of quality characteristics using the Max-MCUSUM control chart with k=0.5. A separate bootstrap UCL was calculated for each combination because the residual dimension and covariance structure differed across subsets. The UCLs were 3.334 for the three-characteristic chart, 3.081 for solution color–grain size, 3.677 for solution color–moisture content, and 1.836 for grain size–moisture content. The table shows that the solution color and grain size combination has the highest number of out-of-control observations. Therefore, it can be concluded that the combination of the quality characteristics of solution color and grain size is suspected to play a role in causing out-of-control observations. These factors include human factors, machines, methods, raw materials, environment, and measurements that affect the color quality characteristics of the solution in the white crystal sugar production process at the Djatiroto Sugar Factory of PT Perkebunan Nusantara XI.

#### Modeling using MLS-SVR

The analysis using MLS-SVR residual-based Max-MCUSUM control charts have several main objectives. Firstly, the method aims to address or reduce autocorrelation in the data. Secondly, this analysis seeks to determine whether the result of the MLS-SVR residual value on the Max-MCUSUM control chart will be more sensitive to shifts than the LSTM model. Lastly, this analysis compares computation time between the LSTM and MLS-SVR methods. The PACF plot is used to get significant lags of each quality characteristic to determine the MLS-SVR model input data. The following is a PACF plot of each quality characteristic of the sugar yield production process at Djatiroto Sugar Factory, as shown in Fig. 7.

**Fig. 7:**
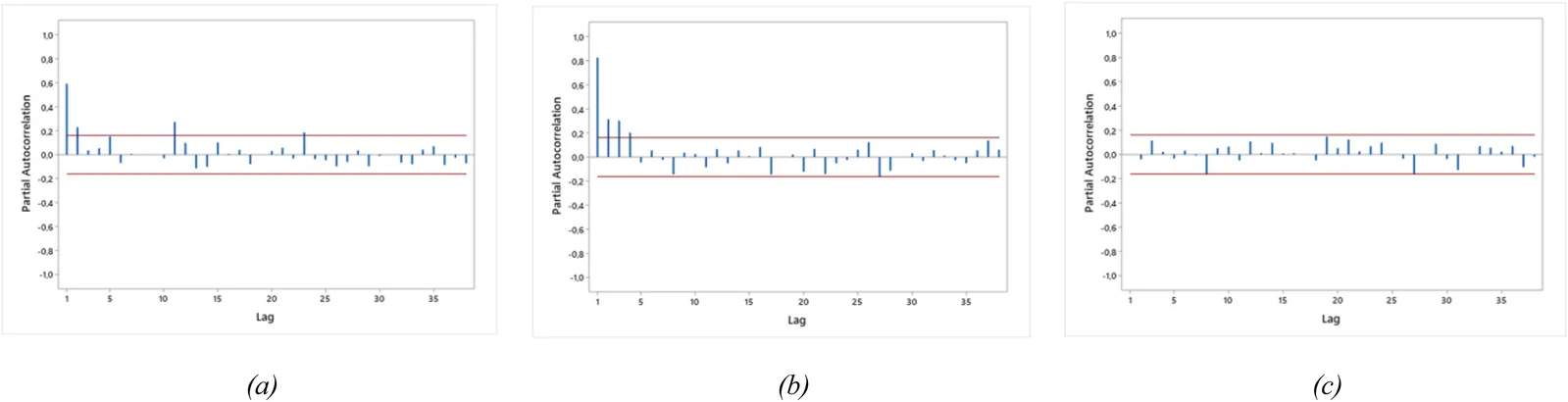
(a) PACF plot of solution color; (b) PACF plot of grain size; (c) PACF plot of moisture content.

Based on Fig. 7, the PACF plot on the quality characteristics of solution color and moisture content in phase I with a significance level of 0.05 has significant PACF plot results on several lags. The MLS-SVR model inputs formed are $y_{1(i-1)},y_{1(i-2)},y_{1(i-3)},y_{1(i-11)},y_{1(i-23)},y_{2(i-1)},y_{2(i-2)},y_{2(i-3)},y_{2(i-4)},y_{2(i-27)},y_{3(i-8)},y_{3(i-27)}$. The MLS-SVR inputs were constructed from the significant PACF-based lags, with the maximum selected lag equal to 27. Consequently, the first 27 Phase I observations could not be used to form complete input vectors. Thus, from the 130 raw Phase I observations, 103 effective observations were available for MLS-SVR modeling and residual monitoring. The selected input is the model that has the minimum MSE value, namely with the optimal hyper-parameter combination $\gamma^{\prime }=2^{20},\;\gamma^{\prime \prime }=2^{20},\;\sigma =2^{20}$ with a minimum MSE value of 0.571. MLS-SVR modeling uses the RBF kernel function with parameters σ.

Several assumptions must be met, namely autocorrelation and multivariate normal distribution. Autocorrelation checking can be done by analysing the Autocorrelation Function (ACF) plot of the phase I residuals. If the ACF plot shows a decrease or approaches zero at certain lags, it can be concluded that autocorrelation has been reduced.

The results of the ACF plots of the three quality characteristics from Fig. 8 show that MLSSVR modeling can reduce autocorrelation in phase I observation data. The MLS-SVR residuals were evaluated using the same practical criteria, where residual autocorrelation was considered acceptable when most ACF values remained within the 95% confidence bounds and no persistent pattern appeared across consecutive lags. It can be seen from the quality characteristics of grain size and moisture content that do not have significant lags, so they do not have autocorrelation. After checking for autocorrelation in the residual data of the MLS-SVR phase I model, a multivariate normal distribution check was carried out on the three quality characteristics of white crystal sugar. Multivariate normality of the Phase I MLS-SVR residuals was assessed using the same multivariate Shapiro–Wilk test. The test yielded W=0.992, so the p-value was 0.116. Obtained that the p-value is greater than α (5%), which means that the residual data in phase I has followed the multivariate normal distribution. Furthermore, process control is carried out using the Max-MCUSUM Control Chart based on the MLS-SVR model residuals.

**Fig. 8:**
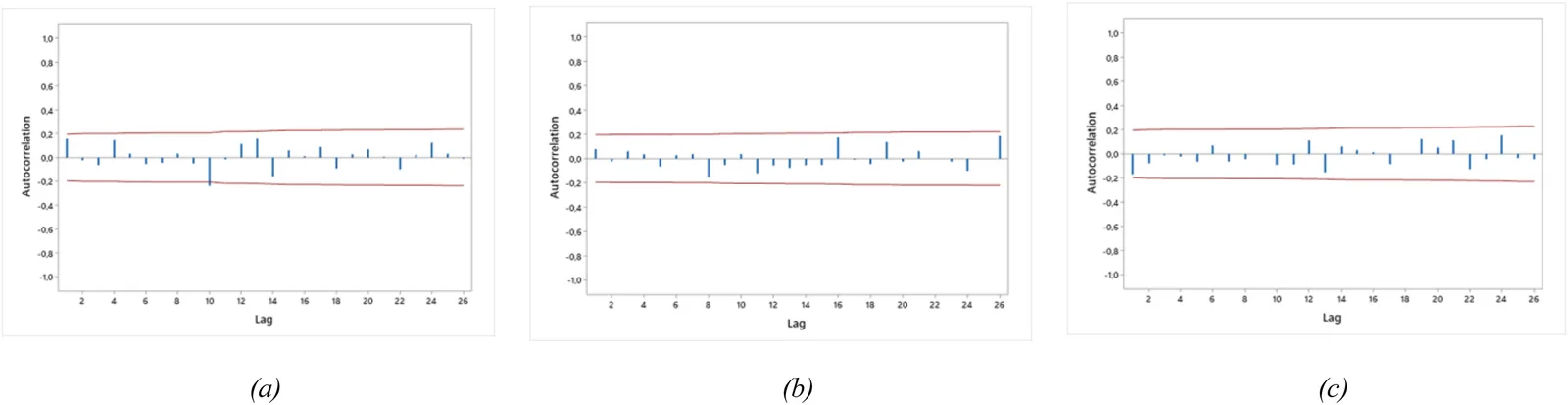
(a) ACF plot of solution color; (b) ACF plot of grain size; (c) ACF plot of moisture content.

#### Phase-I

Fig. 9 (a) shows that the control limit in phase I is 3.850 with k = 0.5 and α = 0.00273. there is one observation outside the control limits, namely the 23rd observation data. which means that the residuals of the MLS-SVR model of the production process of PG Djatiroto's white crystal sugar yield in the 2021 milling season in phase I are still not controlled. the 2021 milling season in phase I is still not statistically controlled and cannot be said to have represented an in-control process. Then in Fig. 9 (b) is the Max-MCUSUM Control Chart based on the residuals of the MLSSVR phase I model, which has been statistically controlled after replacing the first residual value indicated as out of control with the previous residual value, which is in control so that it can be seen that the residuals of the MLS-SVR model phase I of the PG Djatiroto white crystal sugar production process in the 2021 milling season are statistically controlled and can be continued to phase II with a control limit of 3.850 to see the process shift in the production process of PG Djatiroto white crystal sugar products in the 2021 milling season phase II.

**Fig. 9:**
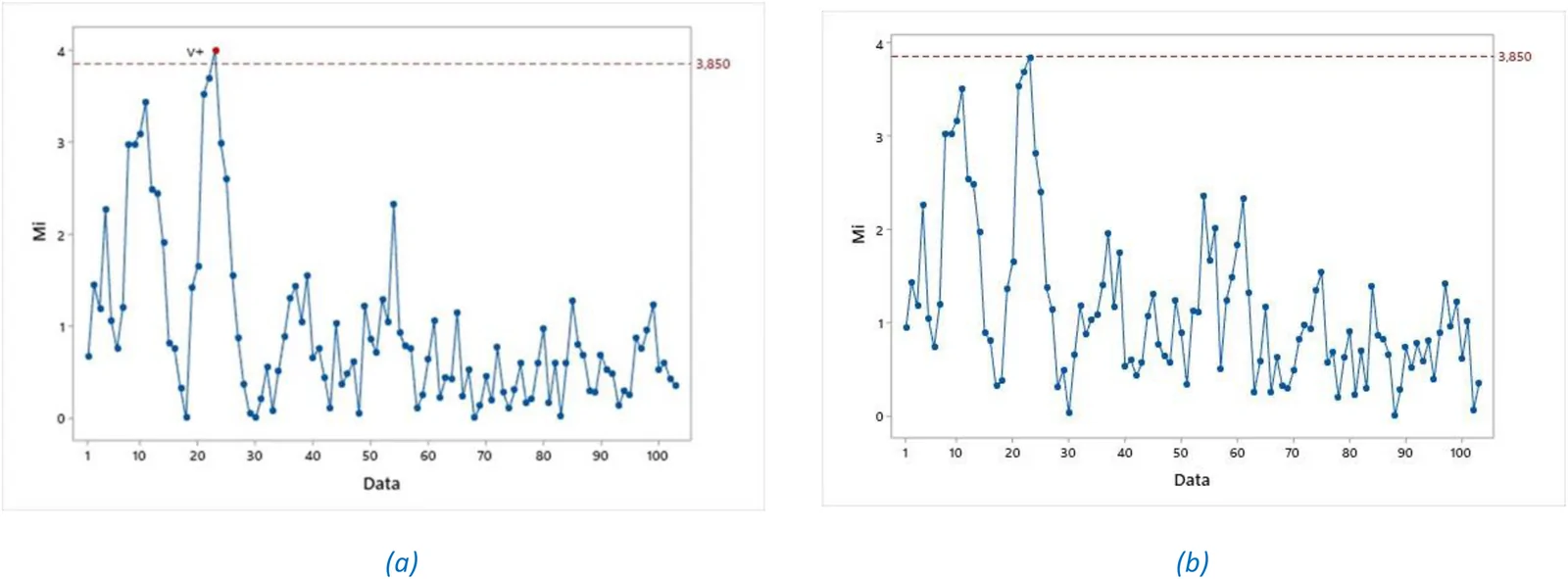
(a) Max-MCUSUM chart based on MLS-SVR residuals phase I; (b) Max-MCUSUM chart based on MLS-SVR residuals phase I in control.

#### Phase-II

Phase II consisted of 21 monitoring observations. Because the required lagged values for the beginning of Phase II were available from the preceding Phase I observations, all 21 Phase II observations could be evaluated using the fitted MLS-SVR model. Based on Fig. 10, the phase II Max-MCUSUM Control Chart with k = 0.5 and control limits of 3.850 has one observation point outside the control limits. So, it can be said that the sugar yield production process at Djatiroto Sugar Factory PT Perkebunan Nusantara XI has not been statistically controlled. The analysis results show that in phase I and II, the production process is not statistically controlled. Therefore, the cause of OOC was identified by considering the combination of two quality characteristic variables: solution color and grain size, solution color and moisture content, and grain size and moisture content. This identification was done using the Max-MCUSUM Control Chart with parameter k = 0.5. The results of out-of-control identification from the Max-MCUSUM Control Chart using the combination of each quality characteristic can be shown in Table 3.

**Fig. 10:**
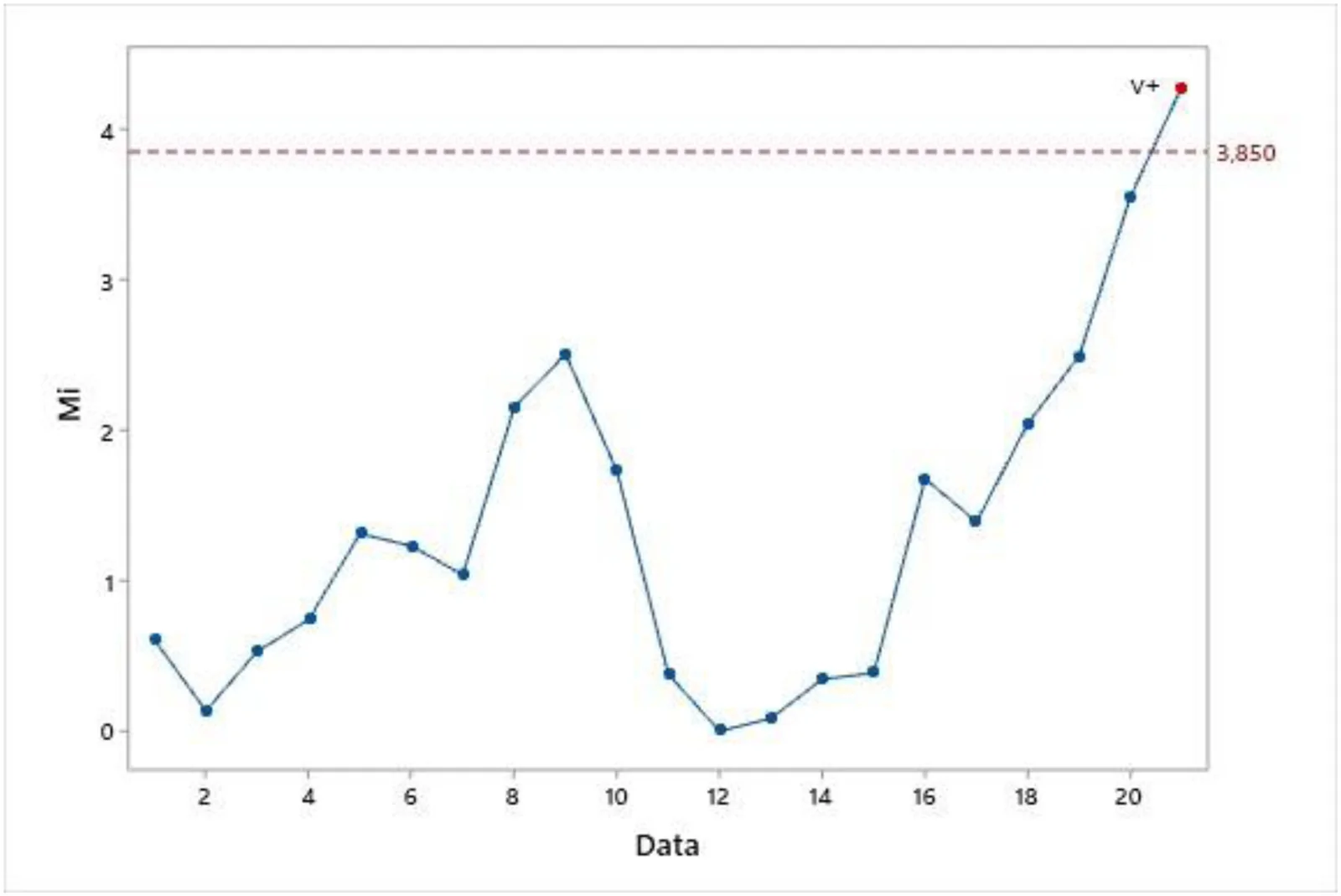
Max-MCUSUM chart based on MLS-SVR residuals phase II.

Following the Phase II monitoring results, the out-of-control signal previously identified during Phase I was further investigated by examining combinations of the quality characteristics. The identification results for the MLS-SVR-based Max-MCUSUM chart are presented in Table 3.

Table 3 shows that the three two-variable combinations, namely solution color–grain size, solution color–moisture content, and grain size–moisture content, each produced three out-of-control observations. These signals indicate potential disturbances in the corresponding quality characteristics during the white crystal sugar production process at the Djatiroto Sugar Factory of PT Perkebunan Nusantara XI and may be associated with production-related factors such as human factors, machinery, methods, raw materials, environmental conditions, and measurement systems.

### Comparison result

To summarize the predictive and monitoring results of the two frameworks, the reported Mean Squared Error (MSE) values and Phase II out-of-control detection results were examined. The LSTM model yielded a training MSE of 0.115, while the minimum MSE reported for the MLS-SVR model was 0.571. In the observed dataset, the LSTM residual-based Max-MCUSUM chart detected three Phase II out-of-control signals, whereas the MLS-SVR-based chart detected one. Each chart used its own bootstrap-derived Phase I control limit, with UCL values of 3.334 for LSTM and 3.850 for MLS-SVR. In addition, the two frameworks were not evaluated using identical effective Phase I sample sizes: the LSTM model used 129 effective observations, whereas the MLS-SVR model used 103 effective observations because its input structure included lags up to 27. Therefore, the difference in the number of detected signals should be interpreted as the observed monitoring outcome under model-specific calibration and effective sample sizes, rather than as evidence of intrinsically greater sensitivity. Both frameworks effectively reduced autocorrelation and supported residual-based monitoring of the production process.

The comparison between the LSTM and MLS-SVR models using the Max-MCUSUM Control Chart showed some important findings. First, the computation time comparison between LSTM and MLS-SVR models may vary depending on the dataset size, model complexity, and specific implementation of the two methods. However, LSTM's higher complexity often requires a longer computation time than MLS-SVR for the same dataset. If computation time is a critical factor in an application, MLS-SVR may be a faster option.

In addition, both managed to reduce autocorrelation in the data well. Both LSTM and MLSSVR models can produce residual values with a low level of autocorrelation, indicating good potential in handling such problems. Then, regarding sensitivity to shifts, the results show that the LSTM model has a slightly more sensitive residual value result than MLS-SVR. Within the Max-MCUSUM control chart, more observations go out of the control limits in the residual value results of the LSTM model compared to MLS-SVR. This indicates that the LSTM model can better detect significant shifts or changes in the data.

Process capability analysis is used to evaluate overall process performance. In this case, a product is considered capable if the production process has been statistically controlled and the resulting product is within the specification limits set by the company. This success can be indicated by an index value greater than one. The M_Cp_ index measures multivariate process capability, which means the extent to which the process has been statistically controlled by considering several quality characteristics simultaneously. Meanwhile, for univariate process capability, the C_p_ and C_Pk_ index are used; this index is used to measure the extent to which the process has been statistically controlled for each quality characteristic separately. When performing the univariate process capability calculation, each quality characteristic in phase I's white crystal sugar production process is evaluated independently.

Table 4 shows that the C_P_ and C_pk_ values for each white crystal sugar production process characteristic have more than one value. quality characteristics of the white crystal sugar production process have more than one value, which means that the white crystal sugar production process at the Djatiroto Sugar Factory PT Perkebunan Nusantara XI has been completed. the white crystal sugar production process at Djatiroto Sugar Factory PT Perkebunan Nusantara XI has been capable, and the process performance is good and capable. Then, we perform a multivariate process capability analysis where the calculation results are in Table 5. However, the capability indices for moisture content should be interpreted with caution because the relatively limited measurement resolution may restrict the observed variability and consequently affect the estimated capability indices.

**Table 4:** Calculation of univariate process capability.

Combination of Characteristics Quality	Cp	Cpk
Solution Color	1.600	1.450
Grain Size	4.520	4.240
Moisture Content	1.820	1.160

**Table 5:** Calculation of multivariate process capability.

Multivariate Process Capability	Value
M_Cp_	2.6462
M_Cpk_	2.2283

Based on Table 5, the above calculations provide information on the M_Cp_ and M_Cpk_ values, where both values are greater than one, which means that the process is capable and multivariate process performance has good precision and accuracy. That way, it can be indicated that the multivariate process performance has good precision and accuracy and is capable.

### Limitations

This study has several limitations that should be considered.

1. **Limited Production Period**
  The analysis is restricted to the sugarcane milling season from May to October 2021. Therefore, the observed process behavior primarily represents the production conditions during this period and may differ under other seasonal or operational conditions.
2. **Fixed Max-MCUSUM Configuration**
  The Max-MCUSUM control chart was implemented using a fixed reference value of \(k=0.5\). Other reference values and control-chart configurations may produce different monitoring characteristics and can be explored in subsequent studies.
3. **Data and Quality-Characteristic Scope**
  This study uses secondary data obtained from the quality control or quality assurance division of PG Djatiroto and focuses on three quality characteristics: solution color, grain size, and moisture content. Future applications may incorporate additional process variables or quality characteristics to provide a broader representation of the sugar production process.
4. **Residual Modeling Approaches**
  This study focuses on LSTM and MLS-SVR as residual modeling approaches within the Max-MCUSUM framework. The two approaches have different modeling characteristics: LSTM captures temporal dependence through its sequential network structure, whereas MLS-SVR uses selected lagged inputs and kernel-based modeling. Their performance is influenced by the selected model architecture and hyperparameter configuration.

For future research, the proposed residual-based Max-MCUSUM framework could be extended by comparing LSTM and MLS-SVR with other time-series and machine-learning approaches, such as GRU, Bidirectional LSTM, XGBoost, or hybrid models. The evaluation may consider predictive performance, autocorrelation reduction, computational efficiency, and process-monitoring performance to provide a broader assessment of alternative residual-generation approaches. Controlled simulation studies with predefined process shifts could also be considered to further investigate the monitoring characteristics of the residual-based Max-MCUSUM framework under different process conditions.

## Ethics statements

This research did not involve research on humans or animals, and no data is involved from social media platforms.

## CRediT authorship contribution statement

**Muhammad Ahsan:** Conceptualization, Methodology, Visualization, Validation**; Muhammad Romiz Rofan Hakim:** Conceptualization, Validation, Software, Methodology, and Visualization; **Syafi’ Bariq’ Syihabuddin Hidayatullah**: Writing – Original Draft and Data Curation; **Muhammad Hisyam Lee**: Writing, Reviewing and Editing.

## Declaration of interests

The authors declare that they have no known competing financial interests or personal relationships that could have appeared to influence the work reported in this paper.

## Data Availability

Data will be made available on request.
