# Supplementary material for: Enhancing multivariate quality monitoring: Integrating LSTM and MLS-SVR with Max-MCUSUM frameworks
Source: MethodsX. 2026 Oct 8;17:104197. doi: 10.1016/j.mex.2026.104197 (PMC13682182; doi:10.1016/j.mex.2026.104197)
Supplement: Supplementary file 1 [file mmc1.pdf]

Python - LSTM Modeling and Data Preprocessing

```
import pandas as pd
import numpy as np
import tensorflow as tf
from sklearn.metrics import mean_squared_error, mean_absolute_error, r2_score
from tensorflow.keras.models import Sequential
from tensorflow.keras.layers import LSTM, Dense, Dropout, BatchNormalization, LeakyReLU,
Add
from tensorflow.keras.optimizers import Nadam
from tensorflow.keras import regularizers
from tensorflow.keras.callbacks import EarlyStopping
import warnings

# Load the Excel file into a Pandas dataframe
data = pd.read_excel('DataTugasAkhir.xlsx', sheet_name='DataStationer')
# Convert the float columns to float datatype
data['BJB'] = data['BJB'].astype(float)
data['KadarAir GKP'] = data['KadarAirGKP'].astype(float)
# Convert the integer column to integer datatype
data['IcumsaGulaProduk'] = data['IcumsaGulaProduk'].astype(float)

# Split the data into train and test set
train = data.iloc[:130,]
test = data.iloc[130:,]

# Transform data to the appropriate shape and scale it
def create_dataset(dataset):
    dataX, dataY = [], []
    for i in range(len(dataset)-1):
        a = dataset[i:(i+1), :]
        dataX.append(a)
        dataY.append(dataset[i+1, :])
    return np.array(dataX), np.array(dataY)

# Scale the data
class RobustMinMaxScaler:
    def __init__(self):
        self.min_value = None
        self.max_value = None
    def fit(self, X):
        self.min_value = np.min(X, axis=0)
        self.max_value = np.max(X, axis=0)
        return self
    def transform(self, X):
        return (X - self.min_value) / (self.max_value - self.min_value)
    def fit_transform(self, X):
        return self.fit(X).transform(X)
```

```
scaler = RobustMinMaxScaler()
scaled_train = scaler.fit_transform(train)
scaled_test = scaler.transform(test)

# Reshape the data
trainX, trainY = create_dataset(scaled_train.values)
testX, testY = create_dataset(scaled_test.values)

# Define the model
model.add(LSTM(512, input_shape=(trainX.shape[1], trainX.shape[2]), return_sequences=True,
recurrent_dropout=0.2, kernel_regularizer=regularizers.l2(0.01)))
model.add(BatchNormalization())
model.add(Dropout(0.4))

# Add Skip Connections with Residuals
lstm1 = LSTM(256, return_sequences=True, recurrent_dropout=0.4,
kernel_regularizer=regularizers.l2(0.01))
lstm2 = LSTM(128, return_sequences=True, recurrent_dropout=0.4,
kernel_regularizer=regularizers.l2(0.01))
lstm3 = LSTM(64, return_sequences=True, recurrent_dropout=0.4,
kernel_regularizer=regularizers.l2(0.01))
lstm4 = LSTM(32, return_sequences=True, recurrent_dropout=0.3,
kernel_regularizer=regularizers.l2(0.01))

lstm_output1 = lstm1(model.layers[-1].output)
residual1 = LSTM(256, return_sequences=True, recurrent_dropout=0.3,
kernel_regularizer=regularizers.l2(0.01))(model.layers[-1].output)
residual1 = Add()([residual1, lstm_output1])

lstm_output2 = lstm2(residual1)
residual2 = LSTM(128, return_sequences=True, recurrent_dropout=0.3,
kernel_regularizer=regularizers.l2(0.01))(residual1)
residual2 = Add()([residual2, lstm_output2])

lstm_output3 = lstm3(residual2)
residual3 = LSTM(64, return_sequences=True, recurrent_dropout=0.3,
kernel_regularizer=regularizers.l2(0.01))(residual2)
residual3 = Add()([residual3, lstm_output3])

lstm_output4 = lstm4(residual3)
residual4 = LSTM(32, return_sequences=True, recurrent_dropout=0.3,
kernel_regularizer=regularizers.l2(0.01))(residual3)
residual4 = Add()([residual4, lstm_output4])

# Final LSTM layer
model.add(LSTM(32, return_sequences=False, recurrent_dropout=0.3,
kernel_regularizer=regularizers.l2(0.01)))
model.add(BatchNormalization())
model.add(Dropout(0.4))

# Additional Dense layers
```

```
model.add(Dense(256, activation='leaky_relu'))
model.add(BatchNormalization())
model.add(LeakyReLU(alpha=0.2))
model.add(Dense(128, activation='leaky_relu'))
model.add(BatchNormalization())
model.add(LeakyReLU(alpha=0.2))
model.add(Dense(64, activation='leaky_relu'))
model.add(BatchNormalization())
model.add(LeakyReLU(alpha=0.2))
model.add(Dense(32, activation='swish'))
model.add(BatchNormalization())
model.add(LeakyReLU(alpha=0.2))
model.add(Dense(trainY.shape[1], activation='linear'))

# Compile the model
optimizer = Nadam(learning_rate=0.001, beta_1=0.9, beta_2=0.999, epsilon=1e-07,
schedule_decay=0.004)
model.compile(loss='mse', optimizer=optimizer)

# Implement Early Stopping
early_stopping = EarlyStopping(monitor='val_loss', patience=10, verbose=1,
restore_best_weights=True)
model.summary()

# Train the model
history = model.fit(trainX, trainY, epochs=200, batch_size=16, validation_split=0.2,
callbacks=[early_stopping])
model.save('lstm_model.h5')

# Predict the values for training and testing datasets
# Calculate residuals for train
train_residuals = trainY - trainPredict
# Calculate residuals for test
test_residuals = testY - testPredict

# Mean squared error (MSE)
mse = mean_squared_error(testY, testPredict)
# Root mean squared error (RMSE)
rmse = np.sqrt(mse)
# Mean absolute error (MAE)
mae = mean_absolute_error(testY, testPredict)
# Coefficient of determination (R^2)
r2 = r2_score(testY, testPredict)

print("Mean Squared Error (MSE):", mse)
print("Root Mean Squared Error (RMSE):", rmse)
print("Mean Absolute Error (MAE):", mae)
print("Coefficient of Determination (R^2):", r2)

# Print residuals
print('Train Residuals:\n', train_residuals)
```

```
print('Test Residuals:\n', test_residuals)
print(trainPredict)
print(testPredict)

import matplotlib.pyplot as plt
# Sample data
x = list(range(1,130))
y1 = trainY[:130,0]
y4 = trainPredict[:130,0]

# Create the figure and axis
fig, ax = plt.subplots()
# Plot the lines with customizations
ax.plot(x, y1, linewidth=2, linestyle='-', color='blue', label='Actual')
ax.plot(x, y4, linewidth=2, linestyle='--', color='red', label='Predicted')

# Set the title and labels with increased fontsize
ax.set_xlabel('Data', fontsize=12)
ax.set_ylabel('Value', fontsize=12)
# Add gridlines
ax.grid(True)
# Add a legend with increased fontsize
ax.legend(fontsize=8)
# Show the plot
plt.show()

import matplotlib.pyplot as plt
from statsmodels.tsa.stattools import acf
data = train_residuals
# Compute and plot autocorrelation for each variable
lags = 128 # Number of lags to consider
plt.figure(figsize=(10,6))
for i in range(data.shape[1]):
   acf_values = acf(data[:,i], nlags=lags)
   plt.stem(range(lags+1), acf_values, markerfmt='C'+str(i), label=f'Variable{i+1}')
plt.xlabel('Lag')
plt.ylabel('Autocorrelation')
plt.title('Autocorrelation Plot for Multivariate Data')
plt.legend()
plt.show()

import matplotlib.pyplot as plt
train_loss = history.history['loss']
val_loss = history.history['val_loss']
epochs = range(1, len(train_loss)+1)
# Plotting the training and validation loss
plt.plot(epochs, train_loss, 'b', label='Training Loss')
plt.plot(epochs, val_loss, 'r', label='Validation Loss')
plt.title('Training and Validation Loss')
plt.xlabel('Epochs')
plt.ylabel('Loss')
```

```
plt.legend()
plt.show()

train_resi_csv = pd.DataFrame(train_residuals)
train_resi_csv.to_csv('train_residual.csv', index=False)
train_predict_csv = pd.DataFrame(trainPredict)
train_predict_csv.to_csv('train_predict.csv', index=False)
print(trainPredict)
print(test_residuals)
test_resi_csv = pd.DataFrame(test_residuals)
test_resi_csv.to_csv('test_residual.csv', index=False)
train_predict_csv = pd.DataFrame(trainPredict)
train_predict_csv.to_csv('train_predict.csv')
test_predict_csv = pd.DataFrame(testPredict)
test_predict_csv.to_csv('test_predict.csv', index=False)
```

MATLAB - Max-MCUSUM Calculation

```
clear;
clc;
load dataf2_ok.mat % the data you use
N = size(X);
n = N(1);
p = N(2);
k = 0.5;
alphas = 0.00273;
xin = mean(X);
sigin = cov(X); % Assuming Xin was a typo for X based on context
sigx = cov(X); % Assuming Xin was a typo for X based on context
xb = mean(X);
lam = sqrt((xb-xin)*(inv(sigin))*(xb-xin)');
for i=1:n
   z(i) = ((xb-xin)*(inv(sigin))*(X(i,:)-xin)')/lam;
end
for qw=1:n
   l(qw) = (X(qw,:)-xin)*(inv(sigx))*(X(qw,:)-xin)';
end
for sa=1:n
   h(sa) = chi2cdf(l(sa),p);
end
for ch=1:n
   if h(ch)==1;
      h(ch)=0.99999;
      w(ch)=norminv(h(ch),0,1);
   else
      w(ch)=norminv(h(ch),0,1);
   end
end
Cp(1)=max(0,(z(1)-k));
Cm(1)=max(0,(-k-z(1)));
Sp(1)=max(0,(w(1)-k));
Sm(1)=max(0,(-k-w(1)));
for j=2:n
   Cp(j)=max(0,(z(j)-(k)+Cp(j-1)));
   Cm(j)=max(0,(-(k)-z(j)+Cm(j-1)));
   Sp(j)=max(0,(w(j)-k+Sp(j-1)));
   Sm(j)=max(0,(-k-w(j)+Sm(j-1)));
end
% kes=[];
for m=1:n
   C(m)=max(Cp(m),Cm(m));
   S(m)=max(Sp(m),Sm(m));
   Mm(m)=max(C(m),S(m));
end
UCL = 4.84534
uclim = max(Mm)+10*UCL/100;
UCLr = repmat(UCL,1,m);
```

```
Xu = (1:m);
plot(Xu,Mm,'-',Xu,UCLr,'r-',Xu,Mm,'bo')
ylim([min(Mm) uclim])
title('Bootstrap-Based Max-MCUSUM Control Chart')
xlabel('Sample')
ylabel('Max-MCUSUM')
```

MATLAB - Max-MCUSUM with Bootstrap

```
clear;
clc;
load '/MATLAB Drive/New Folder/datafase1.mat' % the data you use
N = size(X);
n = N(1);
p = N(2);
k = 0.5;
alphas = 0.00273;
xin = mean(X);
sigin = cov(X);
xb = mean(X);
sigx = cov(X);
lam = sqrt((xb-xin)*(inv(sigin))*(xb-xin)');
for i=1:n
   z(i) = ((xb-xin)*(inv(sigin))*(X(i,:)-xin)')/lam;
end
for qw=1:n
   l(qw) = (X(qw,:)-xin)*(inv(sigx))*(X(qw,:)-xin)';
end
for sa=1:n
   h(sa) = chi2cdf(l(sa),p);
end
for ch=1:n
   if h(ch)==1;
      h(ch)=0.99999;
      w(ch)=norminv(h(ch),0,1);
   else
      w(ch)=norminv(h(ch),0,1);
   end
end
Cp(1)=max(0,(z(1)-k));
Cm(1)=max(0,(-k-z(1)));
Sp(1)=max(0,(w(1)-k));
Sm(1)=max(0,(-k-w(1)));
for j=2:n
   Cp(j)=max(0,(z(j)-(k)+Cp(j-1)));
   Cm(j)=max(0,(-(k)-z(j)+Cm(j-1)));
   Sp(j)=max(0,(w(j)-k+Sp(j-1)));
   Sm(j)=max(0,(-k-w(j)+Sm(j-1)));
end
%kes=[];
for m=1:n
   C(m)=max(Cp(m),Cm(m));
   S(m)=max(Sp(m),Sm(m));
   Mm(m)=max(C(m),S(m));
end
for res=1:200
   boot = randsample(Mm, n, true); % Fixing randsample argument based on typical usage
   UCL1(res) = prctile(boot, 100*(1-alphas));
```

```
end
for res=1:200
    boot = randsample(Mm, 25, true);
    UCL1(res) = prctile(boot, 100*(1-alphas));
end
UCL = 4.84534
uclim = 7
UCLr = repmat(UCL,1,m);
Xu = (1:m);
plot(Xu,Mm,'-',Xu,UCLr,'r-',Xu,Mm,'bo')
ylim([min(Mm) uclim])
title('Bootstrap-Based Max-MCUSUM Control Chart')
xlabel('Sample')
ylabel('Max-MCUSUM')
```

R - Dependency and Normality Testing

```
# DEPENDENCY TEST
library(ggplot2)
library(ggcorrplot)
library(readxl)
data <- read_excel("D:Data.xlsx", sheet=2)
cor_matrix <- cor(data)
ggcorrplot(cor_matrix, type="lower", hc.order = TRUE, lab=TRUE)

cor.test(data$'IcumsaGulaProduk', data$BJB, data$'KadarAirGKP', method="pearson",
alternative="two.sided")
bartlett.test(data)
```

# NORMALITY TEST

```
# Load the required library
library(readxl)
# Read the data from Excel
data <- read_excel("D:FinalData.xlsx", sheet="Data used")
numeric_data <- as.numeric(unlist(data))
# Perform Shapiro-Wilk test
shapiro.test(numeric_data)
```
